# Integrated humoral and inflammatory signatures predict outcomes in severe COVID‐19: a 14‐day longitudinal analysis

**DOI:** 10.1002/cti2.70082

**Published:** 2026-02-24

**Authors:** Hiochelson Najibe Santos Ibiapina, Fabio Magalhães‐Gama, Juliana Costa Ferreira Neves, Fabíola Silva Alves‐Hana, Ismael Artur Costa‐Rocha, Alice Aparecida Lourenço, Ágata Lopes Ribeiro, Geovane Marques Ferreira, Thais Fernanda Campos Fraga‐Silva, Adriana Alves Oliveira Paim, Daisymara Priscila Almeida Marques, Joaquim Pedro Brito‐de‐Sousa, Ana Carolina Campi‐Azevedo, Vanessa Peruhype‐Magalhães, Márcio Sobreira Silva Araújo, Andréa Teixeira‐Carvalho, Vânia Luiza Deperon Bonato, Christiane Becari, Mayra Gonçalves Manegueti, Marcelo Cordeiro‐Santos, Maria Auxiliadora‐Martins, Jordana Grazziela Coelho‐dos‐Reis, Olindo Assis Martins‐Filho, Allyson Guimarães Costa

**Affiliations:** ^1^ Programa de Pós‐Graduação em Medicina Tropical, Universidade do Estado do Amazonas (UEA) Manaus AM Brazil; ^2^ Diretoria de Ensino e Pesquisa Fundação de Medicina Tropical Dr. Heitor Veira Dourado (FMT‐HVD) Manaus Brazil; ^3^ Programa de Pós‐Graduação em Ciências da Saúde, Instituto René Rachou, Fundação Oswaldo Cruz (FIOCRUZ‐Minas) Belo Horizonte Brazil; ^4^ Grupo Integrado de Pesquisas em Biomarcadores, Instituto René Rachou, Fundação Oswaldo Cruz (FIOCRUZ‐Minas) Belo Horizonte Brazil; ^5^ Diretoria de Ensino e Pesquisa, Fundação Hospitalar de Hematologia e Hemoterapia do Amazonas (HEMOAM) Manaus AM Brazil; ^6^ Programa de Pós‐Graduação em Imunologia Básica e Aplicada, Instituto de Ciências Biológicas, Universidade Federal do Amazonas (UFAM) Manaus AM Brazil; ^7^ Hospital Risoleta Tolentino Neves Belo Horizonte Brazil; ^8^ Departamento de Microbiologia, Instituto de Ciências Biológicas Universidade Federal de Minas Gerais (UFMG) Belo Horizonte Brazil; ^9^ Departamento de Bioquímica e Imunologia, Faculdade de Medicina de Ribeirão Preto Universidade de São Paulo Ribeirão Preto Brazil; ^10^ Divisão de Cirurgia Vascular, Departamento de Cirurgia e Anatomia, Faculdade de Medicina de Ribeirão Preto Universidade de São Paulo Ribeirão Preto Brazil; ^11^ Escola de Enfermagem de Ribeirão Preto Universidade de São Paulo Ribeirão Preto Brazil; ^12^ Divisão de Medicina Intensiva, Departamento de Cirurgia e Anatomia, Faculdade de Medicina de Ribeirão Preto Universidade de São Paulo Ribeirão Preto Brazil

**Keywords:** antibodies, biomarkers, cytokines, immunopathogenesis, SARS‐CoV‐2

## Abstract

**Background:**

Severe COVID‐19 is marked by profound immune dysregulation, yet the interplay between humoral and inflammatory responses that determines clinical outcomes in critically ill patients remains incompletely understood. We evaluated longitudinal antibody and soluble immune mediator profiles to identify prognostic signatures associated with survival in severe COVID‐19.

**Methods:**

In this prospective longitudinal study, peripheral blood samples were collected from 30 unvaccinated adults with severe COVID‐19 admitted to the intensive care unit (ICU) and 30 healthy controls. Serum concentrations of SARS‐CoV‐2–specific IgM, IgG and IgA antibodies (S1, RBD and N) and 27 cytokines, chemokines and growth factors were quantified at ICU admission (D0), Day 7 (D7) and Day 14 (D14) using Luminex multiplex assays. Patients were classified according to clinical outcome: discharge (DIS) or death (DEA).

**Results:**

DIS and DEA patients exhibited distinct immunological trajectories. DEA patients showed early elevations of IgM anti‐N and IgM anti‐RBD at D0, accompanied by increased IFN‐γ. At D7, persistently elevated TNF‐α and FGF‐basic differentiated nonsurvivors, while by D14, higher levels of CXCL8, CCL4, CXCL10 and G‐CSF were strongly associated with mortality. In contrast, DIS patients exhibited more coordinated immune regulation, including sustained IL‐13 production and higher IgA anti‐S1 and IgA anti‐RBD levels.

**Conclusions:**

Integrated humoral and inflammatory signatures, particularly early IgM anti‐N/anti‐RBD responses and sequential increases in IFN‐γ, TNF‐α, FGF‐basic, CXCL8, CCL4, CXCL10 and G‐CSF, highlight immune signatures associated with poor outcomes. IL‐13 and coordinated antibody interactions may reflect protective immune pathways. These findings highlight the prognostic value of multidimensional immune monitoring in severe COVID‐19.

## Graphical abstract

Integrated longitudinal profiling of humoral and inflammatory mediators in critically ill COVID‐19 patients revealed distinct immune trajectories associated with clinical outcomes. Nonsurvivors exhibited early IgM‐dominant responses accompanied by persistent inflammatory activation and fragmented immune networks, whereas survivors showed coordinated antibody responses, with IgA anti‐S1 and sustained IL‐13 production, and preserved immune connectivity. These findings highlight the importance of multidimensional immune signatures in defining outcome‐associated trajectories in severe COVID‐19.

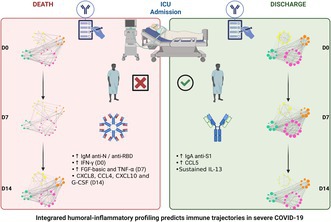

## Introduction

COVID‐19, caused by SARS‐CoV‐2, presents a heterogeneous clinical spectrum ranging from asymptomatic infection to critical illness requiring intensive care support.^1^, ^2^, ^3^, ^4^ Although advances have been made in understanding the pathophysiology of severe disease, the mechanisms driving divergent outcomes among critically ill patients remain only partially understood. Early reports established that dysregulated inflammation—characterised by elevated cytokines, chemokines and growth factors—contributes to pulmonary and systemic injury in severe cases.^5^, ^6^, ^7^, ^8^, ^9^, ^10^, ^11^, ^12^ However, less attention has been directed towards the interplay between this inflammatory milieu and the humoral immune response, particularly the functional dynamics of antigen‐specific antibodies during critical illness.

Antibodies targeting structural SARS‐CoV‐2 proteins, including S1, RBD and N,^13^, ^14^, ^15^, ^16^, ^17^, ^18^, ^19^, ^20^ emerge early during infection and participate not only in neutralisation but also in Fc‐mediated effector functions that regulate cellular activation and inflammation. Evidence suggests that qualitative aspects of the antibody response—such as isotype, subclass distribution and epitope specificity—may be more informative indicators of disease severity than total antibody titres.^21^, ^22^, ^23^, ^24^, ^25^, ^26^, ^27^ These features, however, remain underexplored in critically ill patients, especially in studies evaluating their temporal evolution.

Most investigations assessing humoral immunity in severe COVID‐19 have relied on cross‐sectional designs,^21^, ^22^, ^23^, ^24^, ^25^, ^26^ limiting the understanding of how antibody responses mature during prolonged critical illness. Likewise, few studies have integrated antibody profiles with comprehensive panels of cytokines, chemokines and growth factors to examine how humoral and inflammatory pathways interact over time—an essential step to determine whether specific antibody patterns reflect protective mechanisms or contribute to immune dysregulation.^6^, ^7^, ^9^, ^10^

Brazil experienced intense viral transmission between late 2020 and early 2021, driven predominantly by lineage B.1 before national vaccine rollout.^3^, ^28^, ^29^, ^30^, ^31^ This period provided a unique opportunity to investigate unvaccinated, critically ill patients during sustained viral circulation and high intensive care unit (ICU) burden. Characterising the immune determinants of clinical outcomes in this scenario remains crucial for improving prognostic assessment and guiding therapeutic strategies.

Therefore, this study sought to describe the longitudinal patterns of SARS‐CoV‐2–specific antibodies (IgM, IgG and IgA) directed against S1, RBD, and N, and to evaluate their association with cytokines, chemokines and growth factors in critically ill COVID‐19 patients admitted to intensive care units between October 2020 and March 2021. By integrating humoral and inflammatory profiles at three time points [ICU admission (D0), Day 7 (D7) and Day 14 (D14)], we sought to identify immunological signatures associated with survival or death, providing insights into the influence of antibody responses on the trajectory of severe COVID‐19.

## Results

### Clinical and demographic characteristics according to ICU outcome

A total of 30 critically ill COVID‐19 patients and 30 healthy controls (HC) were included. Table 1 summarises the epidemiological and clinical characteristics of the study population. Most patients were male (70%), although mortality was proportionally higher among females, who accounted for 78% of deaths. The median age did not differ significantly between patients who were discharged (DIS) and those who died (DEA) (61 [51–68] vs. 67 [62–74] years; *P* = 0.111). Hypertension and diabetes mellitus were common in both groups, with no significant between‐group differences (HTN: *P* = 0.079; DM: *P* = 0.228). The SAPS‐3 severity score was also similar between DIS and DEA patients (*P* = 0.799).

**Table 1:** Clinical and sociodemographic data of health controls (HC), DIS and DEA groups

COVID‐19 patients				
Variables	HC, *n* = 30	DIS, *n* = 23	DEA, *n* = 7	*P*‐value
Sex, *n* (%)				
Male	18 (60)	16 (70)	2 (22)	0.153
Female	12 (40)	7 (30)	5 (78)	
Age (years), median [IQR]	67 [59–70]	61 [51–68]	67 [62–74]	0.111
HTN, *n* (%)				
Yes	–	13 (57)	6 (86)	0.079
No	–	10 (43)	1 (14)	
DM, *n* (%)				
Yes	–	15 (65)	4 (57)	0.228
No	–	8 (35)	3 (43)	
HTN + DM, *n* (%)				
Yes	–	10 (43)	4 (57)	0.674
No	–	13 (57)	3 (43)	
COPD, *n* (%)				
Yes	–	–	1 (14)	0.537
No	–	23 (100)	6 (86)	
BMI, median [IQR]	–	30 [26–33]	29 [26–40]	0.224
SAPS3, median [IQR]	–	66 [58–74]	67 [54–85]	0.799

BMI, body mass index; COPD, chronic obstructive pulmonary disease; DM, diabetes mellitus; HTN, hypertension; SAPS3, Simplified Acute Physiology Score III.

### Early and longitudinal humoral responses associated with ICU outcomes

**Figure 1:**
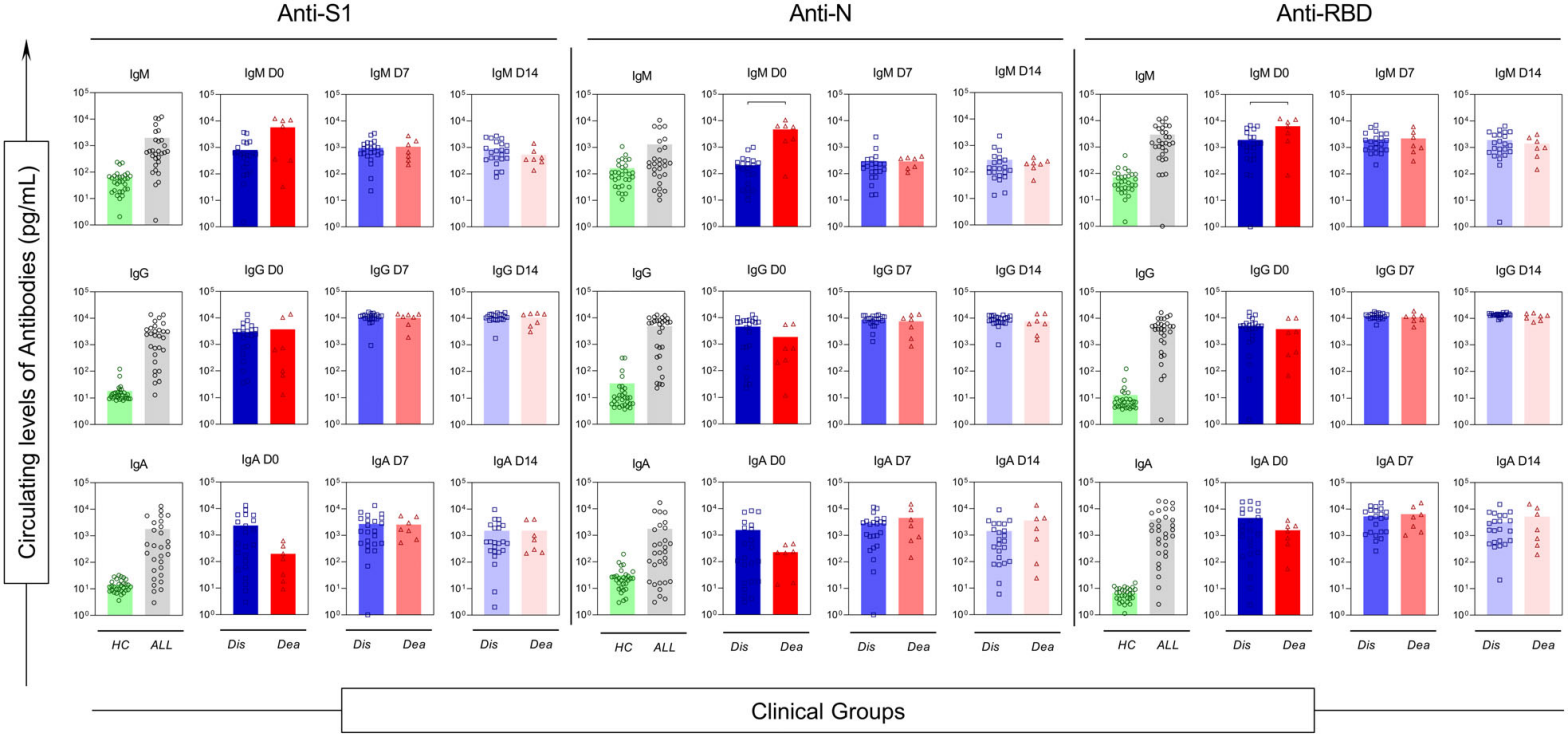
Analysis of the dynamics of soluble immunological molecule production during the clinical evolution of patients (follow‐up) at days D0 (first day in ICU—ALL: 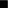; DIS: 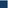; DEA: 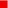), D7 (seventh day in ICU—DIS: 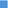; DEA: 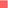), D14 (fourteenth day in ICU—DIS: 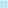; DEA: 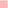) and health controls (HC: 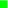). Statistical analyses were performed using the Mann–Whitney test.

To assess the patterns of antibody production in critically ill COVID‐19 patients, serum samples were collected at D0 and on D7 and D14 of hospitalisation, and patients were categorised according to clinical outcome. Figure 1 shows the temporal profiles of IgM, IgG and IgA antibodies. Overall, COVID‐19 patients exhibited higher antibody concentrations than healthy controls (all *P* < 0.05). When stratified by clinical outcome, DIS patients generally displayed higher IgM, IgG and IgA levels over time.

Importantly, at ICU admission (D0), two antibody specificities distinguished clinical outcomes: IgM anti‐N was significantly higher in the DEA group (*P* < 0.05) and IgM anti‐RBD was also elevated among DEA patients (*P* < 0.05). By D7 and D14, antibody titres tended to converge between groups; however, DIS patients maintained higher IgA anti‐S1 and IgA anti‐RBD responses, a finding reinforced in the high‐producer signature analyses.

### Inflammatory immune activation in critically ill COVID‐19 patients compared with healthy controls

**Figure 2:**
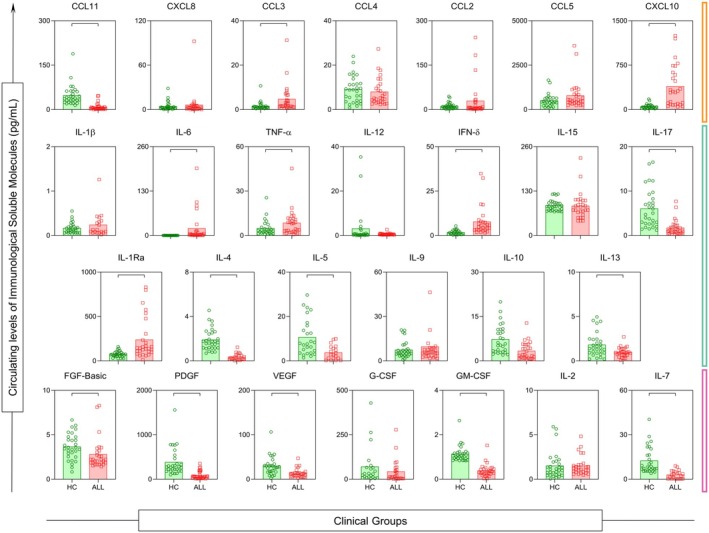
Comparison of the serum concentration values of each molecule in the HC and ALL groups. Significant difference with the HD group *P* < 0.05. Statistical analyses were performed using the Mann–Whitney test.

To further characterise immune activation, we compared cytokine, chemokine and growth‐factor levels between ICU patients (ALL group) and healthy controls (HC), as shown in Figure 2. COVID‐19 patients exhibited significantly higher concentrations of CCL3, CXCL10, IL‐6, TNF‐α, IFN‐γ and IL‐1Ra (all *P* < 0.05), indicating heightened inflammatory activity.

Conversely, healthy controls had higher levels of mediators associated with tissue repair and immunoregulation, including CCL11, IL‐17, IL‐4, IL‐5, IL‐13, FGF‐basic, PDGF, VEGF, GM‐CSF and IL‐7 (all *P* < 0.05). This more regulatory profile contrasts sharply with the inflammatory pattern observed in severe COVID‐19, reflecting pronounced chemokine‐driven leukocyte recruitment and diminished regulatory signalling.

### Temporal evolution of high‐producer immune signatures during ICU stay

**Figure 3:**
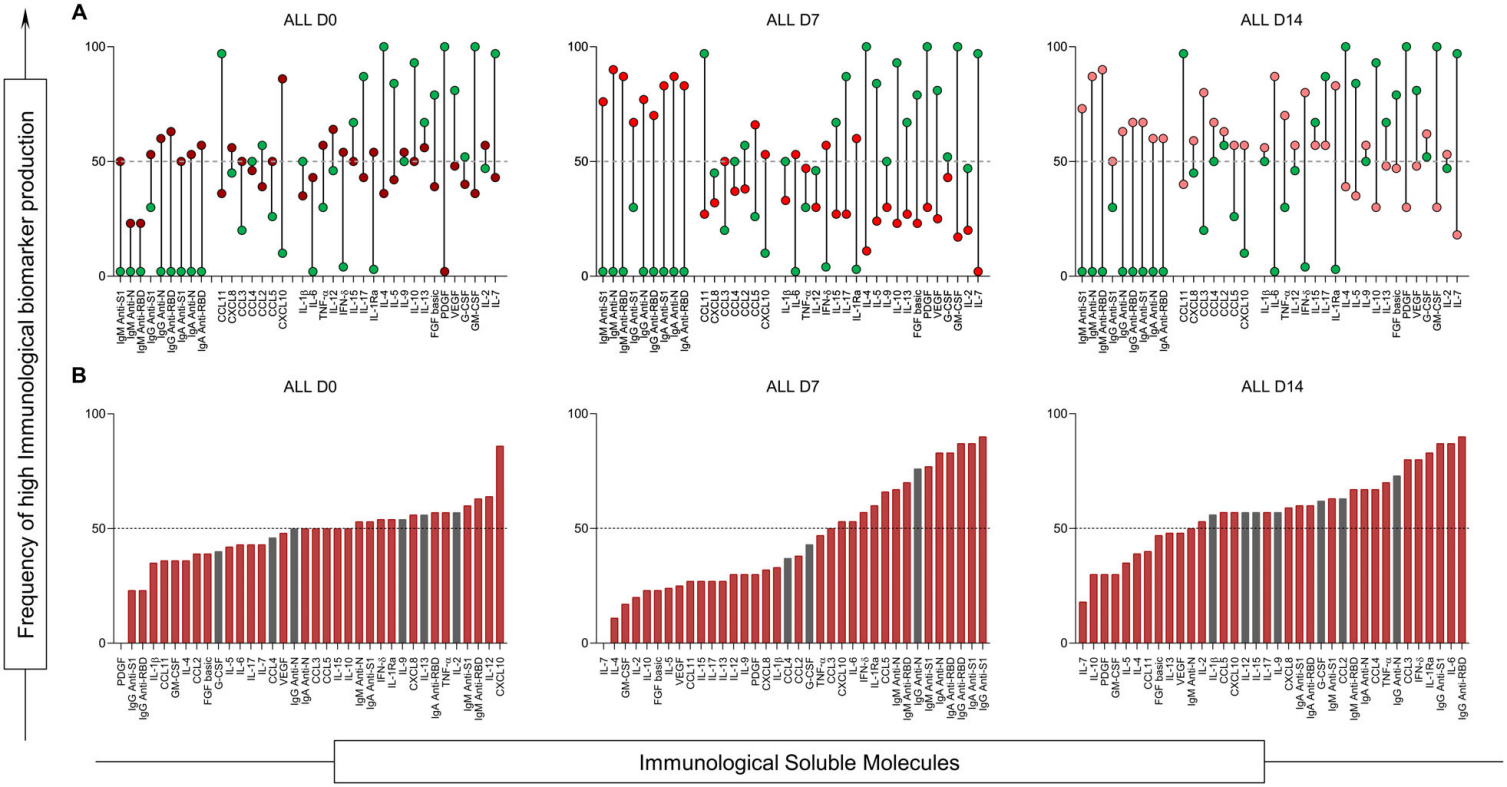
Analysis of high producers is based on the overall median of each molecule. High‐producer status was defined using the global median calculated across all individuals and time points. Bars represent the proportion of individuals classified as high producers for each mediator. These values are converted to percentages, and those molecules with concentrations above 50% (above the dashed line) are considered high producers during the course of the disease on each day of analysis. **(a)** Range of values between the concentrations of molecules in the HC (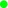) and ALL groups (D0—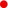; D7—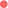; D14—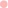); **(b)** The column graph shows the molecules in ascending order of concentration, considering that values above the dashed line indicate highly produced molecules, and those in red (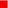) indicate statistically significant differences between the ALL and HC groups. Statistical analyses were performed using the Mann–Whitney test.

To identify dominant immune patterns over time, we applied a global median‐based dichotomisation strategy to define high‐producer signatures of antibodies, cytokines, chemokines and growth factors. Figure 3 presents the high‐producer signature profiles of immune mediators across the ICU follow‐up. At D0, COVID‐19 patients already exhibited high production of several inflammatory molecules. Notably, the number of high‐producer mediators increased progressively from D0 to D14, indicating escalating immune dysregulation over time. Only two mediators—CCL4 and IgG anti‐N—showed no significant differences between ALL and HC groups at any time point, despite being among the most abundant molecules at later stages.

The global median of the molecules was determined based on the concentration values of each patient in each group (HC, DIS and DEA) at each cut‐off point. These values were then converted into percentages, and a 50% cut‐off was established. Individuals showing production above this threshold were classified as high producers.

### Divergent inflammatory trajectories in survivors and nonsurvivors

**Figure 4:**
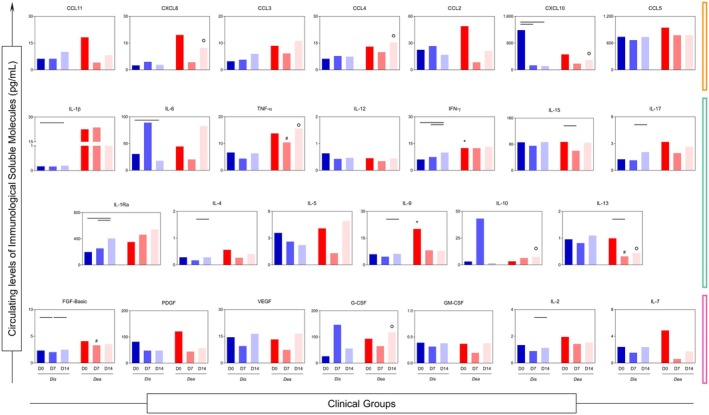
Comparison of the serum concentration values of each molecule in the DIS and DEA groups; Statistical difference between days in group DIS or DEA (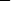); Statistical difference between DIS and DEA groups, considering significance at *P < 0.05 (D0), ^#^ *P* < 0.05 (D7) and ^○^ *P* < 0.05 (D14); Statistical analyses were performed by the Kruskal–Wallis test, followed by Dunn's test in order to compare pairs.

The temporal kinetics of chemokines, cytokines and growth factors differed markedly between DIS and DEA patients (Figure 4). Although considerable heterogeneity was observed across all mediators and time points, some molecules in the DIS group exhibited progressive increases over time, including IFN‐γ, IL‐1Ra and IL‐9 (all *P* < 0.05), although their concentrations remained consistently lower than those observed in the DEA group.

At D7, a shift in production dynamics was noted, with more homogeneous patterns across groups. Nonetheless, TNF‐α and FGF‐basic levels remained significantly elevated in the DEA group (*P* < 0.05). In contrast, DIS patients showed sustained increases in IL‐13 at both D7 and D14, suggesting a potential protective or immunomodulatory role. By D14, DEA patients continued to exhibit high levels of CXCL8, CCL4, CXCL10, TNF‐α, IL‐10 and G‐CSF (all *P* < 0.05 vs. DIS), whereas IL‐13 remained the most distinctive feature associated with survival in the DIS group.

### Outcome‐specific high‐producer immune signatures

The analysed groups of molecules follow the colour scheme and order presented in the legend, according to their respective starting points (1, 10, 17 and 30). Node size is proportional to the number of correlations established: the larger the node, the greater the number of interactions. Similarly, line thickness represents the strength of the correlations, such that stronger correlations are depicted by thicker lines.

**Figure 5:**
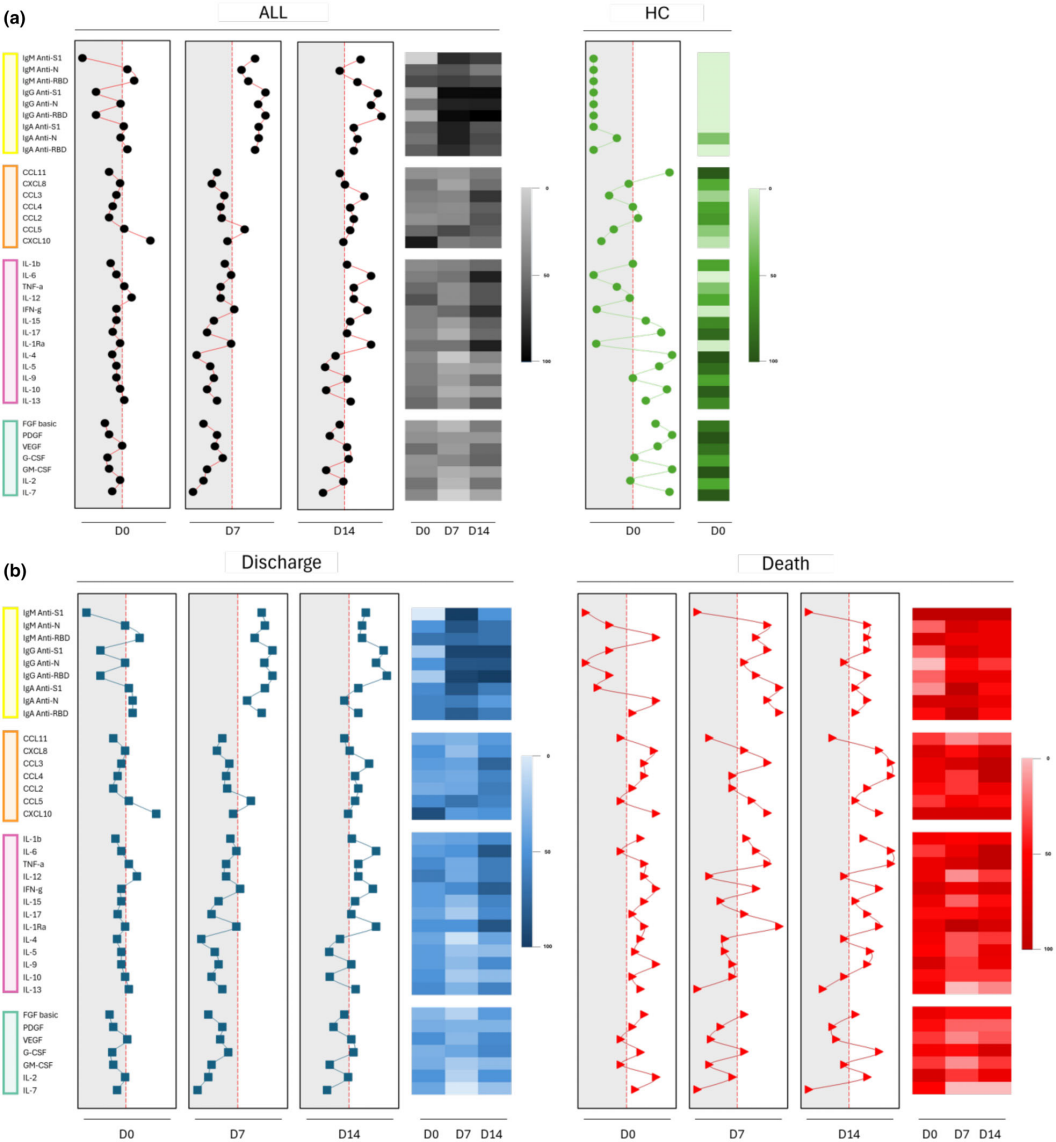
Molecules with concentrations above 50% (right side the dashed line) are considered high producers during the course of the disease on each day of analysis. The graph shows concentration for each function group of (antibodies, chemokines, cytokines and growth factors), considering that values right the dashed line indicate highly produced and left dashed line indicate low produced in ALL (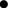), HC (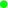), DIS (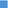) and DEA (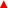) groups. Considering that the cut‐off point is based on the overall skein of each molecule.

Figure 5 summarises the distribution of high‐producer immune mediators among DIS and DEA patients. In the DIS group, high‐producer signatures were dominated by IgA anti‐S1, IgA anti‐RBD and CCL5 (Figure 5a). In contrast, the DEA group consistently exhibited high production of a broader inflammatory repertoire across all time points (D0, D7 and D14), including IgM anti‐N, IgM anti‐RBD, CXCL8, CXCL10, CCL3, IL‐1β, TNF‐α, IFN‐γ, IL‐17, IL‐1Ra and FGF‐basic (Figure 5b). These patterns reinforce the divergence in immune trajectories associated with clinical outcome.

### Outcome‐associated differences in immune coordination revealed by integrative network analysis

**Figure 6:**
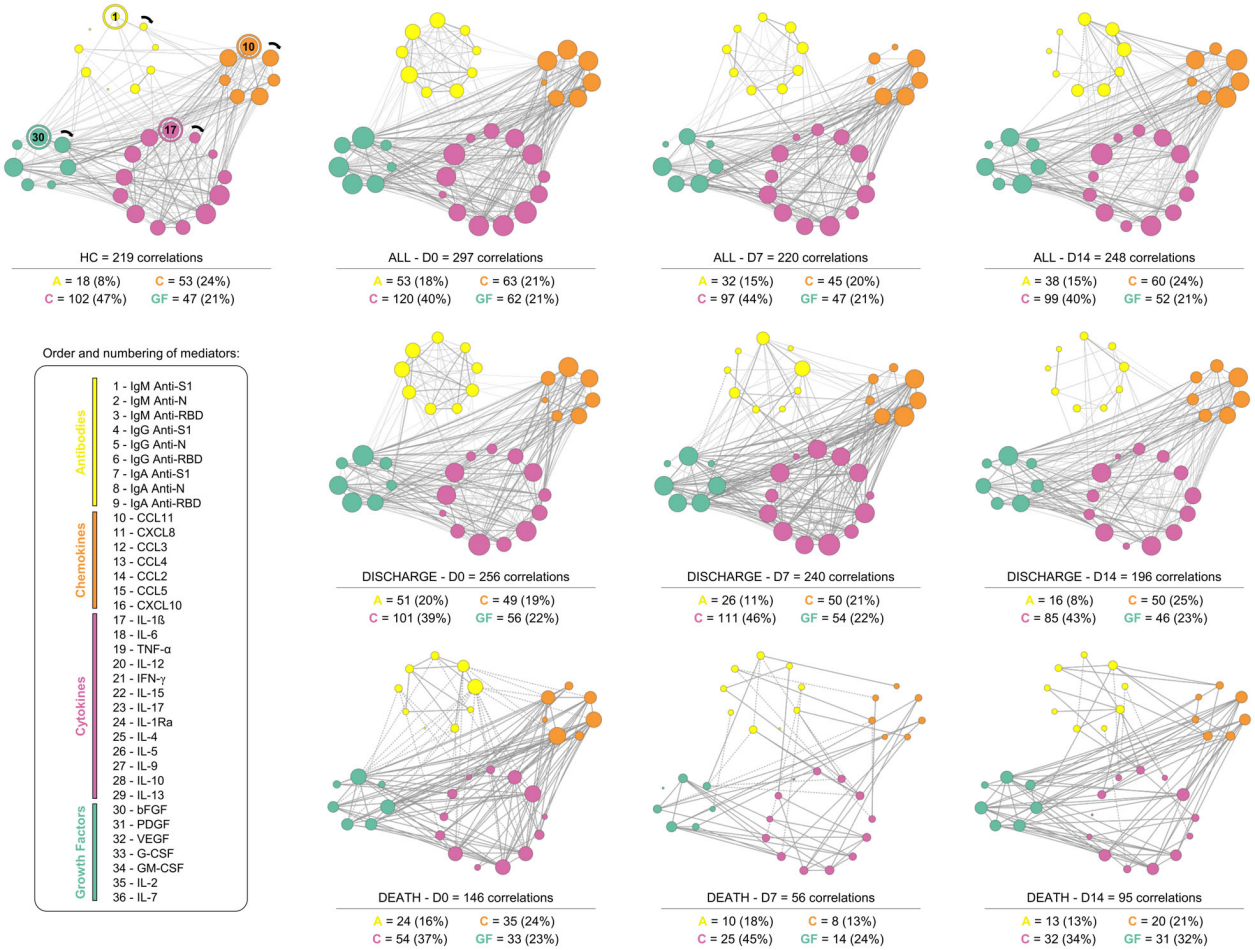
Integrative network correlation analysis of Soluble Immune Mediators of groups HC, ALL, DIS and DEA. Nodes represent clusters of antibodies (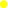), chemokines (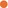), cytokines (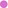) and growth factors (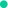). Solid lines indicate significant positive correlations; dashed lines are negative correlations, with varying thickness for strength. The correlation index (*r*) was used to categorise the correlation strength as weak (*r* ≤ 0.35), moderate (*r* ≥ 0.36 to *r* ≤ 0.67) or strong (*r* ≥ 0.68).

Integrative correlation networks were used to explore the coordination between humoral and inflammatory immune mediators over time rather than to infer causality. In this context, network density and connectivity reflect the number and strength of statistically significant correlations among immune components, providing a qualitative representation of immune coordination during critical illness. Changes in network structure across hospitalisation days and between outcome groups were interpreted as shifts in immune integration or fragmentation over time. The biological interaction networks between antibodies, cytokines, chemokines and growth factors provide an integrated view of immune coordination across hospitalisation (Figure 6).

In ALL patients, strong intra‐antibody correlations were observed at D0, accompanied by predominantly negative correlations between antibodies and inflammatory mediators. Over time, these relationships shifted gradually towards more positive interactions.

When comparing DIS and DEA networks, DIS patients exhibited markedly greater overall network connectivity, including stronger antibody–cytokine and antibody–chemokine interactions, along with a progressive and orderly reduction in network density from D0 to D14—suggesting controlled immune resolution. In contrast, DEA patients displayed fewer interactions overall, with early loss of antibody connectivity (particularly IgA and IgG) and an abrupt, disorganised decline in network density throughout hospitalisation.

These findings indicate that preserved coordination between humoral and inflammatory pathways may contribute to survival, whereas disruption of network integration appears to characterise fatal outcomes.

## Discussion

Severe COVID‐19 is characterised by an exacerbated inflammatory response and multisystem dysfunction that frequently leads to the need for invasive ventilatory support and ICU admission. Consistent with previous reports, most patients in our cohort presented comorbidities such as hypertension and diabetes mellitus, conditions widely associated with unfavorable outcomes.^32^, ^33^, ^34^, ^35^, ^36^, ^37^ Although chronic obstructive pulmonary disease (COPD) has also been described as a risk factor for worsening respiratory function, only one patient in our study presented this comorbidity, reinforcing prior observations that severe COVID‐19 itself is often the primary determinant of ICU admission, independent of baseline comorbidities.^38^, ^39^

Despite its established use as a severity index, SAPS‐3 did not differ significantly between DIS and DEA groups, suggesting limited discriminatory power in this cohort. This aligns with findings from Basiri et al.,^40^ who proposed that other tools, such as the SOFA score, may better capture dynamic physiological changes in critically ill COVID‐19 patients.

Following SARS‐CoV‐2 infection, the host mounts an innate immune response,^41^, ^42^ which is subsequently complemented by the humoral response involving antibody production, particularly IgM, IgG and IgA isotypes.^43^ Several studies have highlighted heterogeneity in the magnitude and specificity of antibody responses, influenced by factors, such as disease severity, antigenic targets and timing of seroconversion.^38^, ^44^, ^45^, ^46^, ^47^, ^48^ Both S and N proteins are considered immunodominant in coronaviruses, and the RBD region of the S1 subunit is particularly relevant because of its central role in viral entry.^49^, ^50^, ^51^, ^52^

In our cohort, IgM, IgG and IgA concentrations were similar between DIS and DEA groups on D7 and D14, corroborating observations from Salgado et al.^26^ suggesting that the humoral response in severe COVID‐19 may be globally impaired and not strongly associated with clinical outcome. Nonetheless, at D0, DIS patients showed a trend towards higher IgA and IgG concentrations, which could reflect earlier or more coordinated humoral activation. Elevated IgA levels have also been described in patients with gastrointestinal manifestations of COVID‐19,^53^ although this pattern was not specifically assessed in our population. Interestingly, IgA levels remained stable throughout hospitalisation in our cohort, diverging from studies suggesting a rapid decline in IgA compared with IgG.^1^, ^54^, ^55^, ^56^ While Salgado et al.^26^ associated aberrant IgG responses with mortality,^53^ we did not observe such differences. Variability in patient‐specific factors—including disease duration, viral load and immunological fitness—may account for these divergences.

Upon examining baseline (D0) immune signatures, we found that IgM anti‐N and IgM anti‐RBD were significantly elevated in the DEA group. Such early increases in IgM may reflect uncontrolled viral replication or dysregulated humoral activation, aligning with observations that early, excessive IgM responses can be associated with poor prognosis.^39^ In contrast, IgM anti‐S1 displayed broad interactions with other immune mediators among DIS patients, suggesting a potentially beneficial regulatory or neutralising effect.

The observation that elevated IgM anti‐N and IgM anti‐RBD levels at ICU admission were associated with mortality warrants careful interpretation. Rather than reflecting an effective antiviral response, early IgM dominance may indicate persistent viral antigen exposure, delayed or impaired class‐switch recombination, or dysregulated humoral maturation, phenomena that have been described in severe COVID‐19.^6^, ^26^, ^27^ Previous studies have shown that critically ill patients may exhibit prolonged or uncoordinated IgM responses that fail to transition effectively towards IgG and IgA production, reflecting broader immune dysregulation rather than protection^6^, ^27^. In this context, early IgM predominance, particularly when accompanied by heightened inflammatory activity, may represent a marker of maladaptive humoral activation within a globally dysregulated immune landscape.

The inflammatory response also diverged significantly between clinical outcomes. As extensively reported, cytokine dysregulation, including increases in IL‐6, TNF‐α, IL‐1β and IFN‐γ, is a hallmark of severe COVID‐19.^57^, ^58^, ^59^ These mediators contribute to endothelial dysfunction and vascular injury,^60^, ^61^ supporting the rationale for integrating organ dysfunction scores, such as SOFA into prognostic evaluation.^40^ In our study, DEA patients consistently exhibited higher levels of inflammatory molecules, particularly IFN‐γ, IL‐9, TNF‐α and FGF‐basic. Elevated IFN‐γ and TNF‐α have been associated with extensive tissue damage in SARS‐CoV‐1 and MERS‐CoV infections,^28^, ^29^, ^30^, ^31^, ^62^, ^63^ and our findings reinforce their relevance in COVID‐19–related respiratory deterioration. High IL‐10 in DEA patients likely reflects a compensatory but insufficient attempt to counterbalance the cytokine storm.

Chemokines such as CXCL8 and CXCL10 were also markedly elevated in DEA patients, consistent with their association with neutrophil recruitment, lung injury and severe bilateral pneumonia.^4^, ^29^, ^64^ These findings highlight a strongly pro‐inflammatory chemokine axis that persists and intensifies in patients with fatal outcomes.

IL‐13 emerged as a distinguishing mediator among DIS patients, remaining elevated at D7 and D14. Although IL‐13 has been implicated in type 2 immune polarisation, airway remodelling and mucus hypersecretion, particularly in chronic respiratory diseases,^65^, ^66^ its role in acute viral infections appears to be context‐dependent. Experimental evidence suggests that IL‐13 may reduce ACE2 expression and enhance epithelial barrier and mucus‐associated defences, potentially limiting viral entry and tissue damage.^67^ In this context, the association of sustained IL‐13 production with survival in our cohort likely reflects an immunoregulatory milieu rather than a direct antiviral effect. Thus, IL‐13 may act as a marker of balanced immune modulation during critical illness, highlighting the importance of immune coordination rather than the isolated effect of individual cytokines.

Network analysis highlighted striking differences in immune coordination between DIS and DEA groups. DIS patients exhibited dense and structured networks, including strong correlations among IgM, IgG and IgA antibodies and negative associations with cytokines, such as IL‐6, TNF‐α and IFN‐γ. These patterns suggest an orchestrated immune interaction aimed at containing inflammation while maintaining antibody‐mediated viral control. Positive correlations between IgA anti‐S1 and VEGF further suggest processes associated with endothelial repair or mucosal defence.^60^, ^68^, ^69^, ^70^, ^71^ Conversely, DEA patients displayed sparse networks with early loss of antibody connectivity, particularly IgA and IgG, and disorganised inflammatory interactions that deteriorated over time. These features are compatible with immune exhaustion and loss of coordinated host defence, which have been associated with poor outcomes in other studies.

At D14, DEA patients exhibited a profile resembling the D7 DIS network but with a shift towards maladaptive antibody patterns (IgM anti‐N, IgA anti‐N and IgG anti‐S1), reinforcing the notion of delayed or dysfunctional humoral maturation. These findings highlight the importance of temporal immune coordination rather than isolated biomarker levels in determining disease trajectory.

The correlation network analyses are intended as exploratory and descriptive representations of immune coordination rather than causal models. Because these analyses are based on unadjusted correlations, they do not account for potential confounding by demographic or clinical variables, such as age, sex, BMI or disease severity. Given the limited sample size, particularly among nonsurvivors, and the longitudinal structure of the data, adjusted or regression‐based models were not performed to avoid overfitting and unstable estimates. Accordingly, differences in network density and immune signatures should be interpreted as qualitative, hypothesis‐generating indicators of immune integration that warrant validation in larger cohorts.

An additional consideration relates to the epidemiological context of this study. All patients were unvaccinated and infected during early pandemic waves dominated by the SARS‐CoV‐2 B.1 lineage. In contemporary settings characterised by widespread vaccination and hybrid immunity, baseline humoral and inflammatory states differ substantially and may alter immune trajectories and outcome‐associated signatures. Therefore, the immune patterns identified here should be interpreted within their historical context. Despite these limitations, the prospective design, longitudinal sampling, standardised ICU management and integrated analysis of humoral and inflammatory responses provide relevant insights into immune coordination in severe COVID‐19.

## Conclusion

In this prospective 14‐day longitudinal analysis, we demonstrated that critically ill COVID‐19 patients exhibit marked dysregulation of humoral and inflammatory responses, with distinct immune signatures associated with survival or death. Early elevations of IgM anti‐N and IgM anti‐RBD at ICU admission, accompanied by increased IFN‐γ, characterised nonsurvivors and were followed by persistent inflammatory activation involving TNF‐α, FGF‐basic, CXCL8, CCL4, CXCL10 and G‐CSF across hospitalisation.

Conversely, survivors exhibited more coordinated immune responses, including sustained IL‐13 production and higher IgA anti‐S1 and IgA anti‐RBD levels, suggesting an immunomodulatory milieu rather than isolated inflammatory activation. Integrative network analyses reinforced these findings, revealing that preserved interactions among antibodies, cytokines, chemokines and growth factors were hallmarks of survival, whereas progressive loss of immune coordination characterised fatal outcomes.

Taken together, these findings indicate that integrated humoral–inflammatory signatures, rather than isolated biomarker measurements, provide meaningful insights into immune trajectories associated with clinical outcomes in severe COVID‐19. While these signatures should be interpreted as exploratory and hypothesis‐generating, they highlight the potential of multidimensional immune profiling to inform patient stratification and immune monitoring in future studies, pending validation in larger and contemporary cohorts.

## Material and methods

### Study design and setting

**Figure 7:**
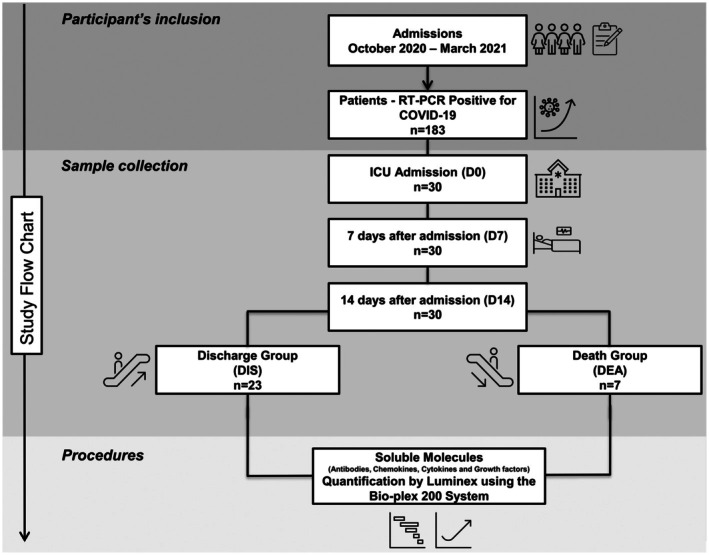
Flowchart illustrating selection of the 30 participants.

This prospective, longitudinal observational study enrolled critically ill adults with confirmed COVID‐19 admitted to the ICUs of Hospital Risoleta Tolentino Neves (Belo Horizonte, MG, Brazil) and Hospital das Clínicas da Faculdade de Medicina de Ribeirão Preto, Universidade de São Paulo (Ribeirão Preto, SP, Brazil). Recruitment occurred between October 2020 and March 2021, during circulation of the SARS‐CoV‐2 B.1 lineage and before the start of COVID‐19 vaccination in Brazil. The study followed STROBE guidelines for observational cohorts, and a flowchart illustrating patient selection is presented in Figure 7.

### Participants

Participants considered eligible for the study fulfilled all of the following requirements: age ≥ 18 years; SARS‐CoV‐2 infection verified by RT‐qPCR; severe COVID‐19 necessitating ICU admission; and availability of serum samples collected at D0 and during follow‐up (D7 and/or D14). Severe COVID‐19 was characterised in accordance with national guidelines, encompassing acute respiratory failure requiring invasive or non‐invasive ventilatory support, PaO_2_/FiO_2_ < 200 mmHg, hemodynamic instability or shock (systolic blood pressure < 90 mmHg or mean arterial pressure < 65 mmHg), and severe acidosis (pH ≤ 7.35 with PaCO_2_ ≥ 50 mmHg). Furthermore, all patients received standardised ICU management, including systemic corticosteroid therapy (methylprednisolone at a dose of 1 mg/kg), prophylactic anticoagulation with enoxaparin (40 U administered subcutaneously once daily) and empirical antibiotic therapy, which was subsequently adjusted according to blood culture results. None of the patients received antiviral therapy during their ICU stay. Exclusion criteria comprised pregnancy, active malignancy, primary or secondary immunodeficiency, autoimmune or rheumatologic disease, chronic use of immunosuppressive therapy, solid organ transplantation and absence of follow‐up serum samples. Noteworthy, information on the use of systemic corticosteroids prior to ICU admission was not available in the medical records; therefore, no exclusions were made based on pre‐admission corticosteroid exposure. Healthy controls were recruited during the same period and had no history of COVID‐19–related symptoms and a negative RT‐qPCR test at recruitment. As serological testing was not systematically performed, prior asymptomatic infection cannot be completely excluded.

### Clinical data collection

Demographic and clinical information was obtained from electronic medical records at baseline and throughout the ICU stay, including age, sex, BMI, comorbidities (hypertension, diabetes mellitus, COPD and others), SAPS‐3 score and clinical outcome (discharge or death).

### Outcomes

The primary outcome of the study was ICU mortality, defined as death occurring at any time during ICU hospitalisation. The secondary outcome was ICU discharge, with patients being followed until either discharge or death.

### Sampling strategy

A non‐probabilistic convenience sample was used because of constraints imposed by the pandemic peak. Peripheral blood (6 mL) was collected at three time points: D0, D7 and D14. Samples were centrifuged, aliquoted and kept at −80°C. Prior to the quantification of all immune molecules at the Instituto René Rachou, Fundação Oswaldo Cruz (FIOCRUZ‐Minas), serum samples were thawed, subjected to centrifugation at 24 000 × *g* and passed through a 0.45 μm filter membrane to eliminate debris. Importantly, the limited sample size and the uniformity of treatment and exposure across all patients mitigate potential biases that could otherwise influence variability in the observed immune response patterns.

### Measurement of SARS‐CoV‐2–specific antibodies

IgM, IgG and IgA antibodies targeting S1, RBD and N antigens were quantified using the Bio‐Plex Pro™ Human IgM SARS‐CoV‐2/RBD/S1/N/4‐plex Kit (Lot: 64399243), the Bio‐Plex Pro™ Human IgG SARS‐CoV‐2/RBD/S1/N/4‐plex Kit (Lot: 64420111) and the Bio‐Plex Pro™ Human IgA SARS‐CoV‐2/RBD/S1/N/4‐plex Kit (Lot: 64420108) (Bio‐Rad Laboratories, Hercules, CA, USA). Antibody levels were acquired on the Luminex 200™ system and analysed using the Bio‐Plex Manager™ software.

### Measurement of soluble immune mediators

Chemokines (CXCL8, CXCL10, CCL2, CCL3, CCL4, CCL5 and CCL11), cytokines (IL‐1β, IL‐1Ra, IL‐2, IL‐4, IL‐5, IL‐6, IL‐7, IL‐9, IL‐10, IL‐12, IL‐13, IL‐15, IL‐17, IFN‐γ and TNF‐α) and growth factors (G‐CSF, GM‐CSF, FGF‐basic and VEGF) were quantified using the Bio‐Plex Pro™ Human Cytokine 27‐plex Assay Kit (Lot: 64480554). Data acquisition was performed on the Luminex 200™ platform, and concentrations were processed using the Bio‐Plex Manager™ software.

### Data analysis

Statistical analyses were conducted using GraphPad Prism v8.0.2 and Stata v13.0. Data normality was tested using the Shapiro–Wilk test. Comparisons between two groups were performed using the Mann–Whitney test, and comparisons involving three or more groups were assessed using the Kruskal–Wallis test followed by Dunn's post‐test for multiple comparisons. For signature analyses, continuous concentrations of antibodies and soluble immune mediators were converted into categorical variables based on a median‐based dichotomisation strategy. Median thresholds were calculated globally using values from all individuals (healthy controls and COVID‐19 patients) and all time points combined. Individuals with values above the global median were classified as high producers, whereas those with values at or below the median were classified as low producers. Fisher's exact test was used to determine statistical significance. Lollipop charts were generated to visualise high‐producer signatures. Integrative correlation networks between antibodies, cytokines, chemokines and growth factors were constructed using Spearman's correlation coefficients and visualised in Cytoscape v3.10.1 (Cytoscape Consortium, San Diego, CA, USA). Statistical significance was defined as *P* < 0.05.

### Ethical issues

All participants provided written informed consent. The study protocol was approved by the Ethics Committee at Instituto René Rachou‐FIOCRUZ‐Minas (CAAE: 42560721.7.0000.5091) and by the Ethics Committee of Hospital das Clínicas da Faculdade de Medicina de Ribeirão Preto—Universidade de São Paulo (CAAE: 30816620.0.0000.5440). All procedures complied with the Declaration of Helsinki and Brazilian Resolution No.: 466/2012.

## Author contributions

**Hiochelson Najibe Santos Ibiapina:** Conceptualisation; investigation; writing – original draft; writing – review and editing; data analysis; curation and visualisation. **Fabio Magalhães‐Gama** and **Ismael Artur Costa‐Rocha:** Data analysis; curation and visualisation. **Juliana Costa Ferreira Neves** and **Fabíola Silva Alves‐Hana:** Curation and visualisation. **Alice Aparecida Lourenço**, **Ágata Lopes Ribeiro**, **Geovane Marques Ferreira**, **Thais Fernanda Campos Fraga‐Silva**, **Adriana Alves Oliveira Paim**, **Daisymara Priscila Almeida Marques**, **Joaquim Pedro Brito‐de‐Sousa**, **Márcio Sobreira Silva Araújo**, **Vânia Luiza Deperon Bonato**, **Christiane Becari**, **Mayra Gonçalves Manegueti** and **Maria Auxiliadora‐Martins:** Conceptualisation; investigation and methodology. **Ana Carolina Campi‐Azevedo**, **Vanessa Peruhype‐Magalhães** and **Jordana Grazziela Coelho‐dos‐Reis:** Methodology; curation and visualisation. **Andréa Teixeira‐Carvalho:** Conceptualisation; supervision; funding acquisition; resources; validation. **Marcelo Cordeiro‐Santos:** Conceptualisation; supervision and coordination; investigation; writing – review and editing. **Olindo Assis Martins‐Filho:** Conceptualisation; supervision and coordination; investigation; funding acquisition; resources; writing – review and editing. **Allyson Guimarães Costa:** Conceptualisation; writing – original draft; writing – review and editing; data analysis; curation and visualisation. All authors read and approved the final version of the manuscript.

## Conflict of interest

The authors declare no conflict of interest.

## Data Availability

The datasets generated during and/or analysed during the current study are available from the corresponding author upon reasonable request.
